# The effect of burden and loneliness on depression in female caregivers through purpose in life

**DOI:** 10.1186/s12889-025-25882-4

**Published:** 2025-12-08

**Authors:** Inmaculada Aragonés Barberá, Alejandro Sanchis-Sanchis, Catalina Patricia Morales-Murillo, María Dolores Grau Sevilla, Familia y factores de riesgo y protección en discapacidad y psicopatología. Campus Capacitas

**Affiliations:** 1 Facultad de Psicología, Departamento Ciencias de la Ocupación., Universidad Católica de Valencia San Vicente Mártir, Av. de la Ilustración, 4, Burjassot, Valencia, 46100 Spain; 2 Facultad de Psicología. Departamento Psicología de la Personalidad, Tratamiento y Metodología., Universidad Católica de Valencia San Vicente Mártir, Av. de la Ilustración, 4, Burjassot, Valencia, 46100 Spain; 3 Facultad de Psicología. Departamento de Psicología Básica, Neuropsicología y Social., Universidad Católica de Valencia San Vicente Mártir, Av. de la Ilustración, 4, Burjassot, Valencia, 46100 Spain; 4 Facultad de Psicología, Universidad Católica de Valencia San Vicente Mártir, Av. de la Ilustración, 4, Burjassot, Valencia, 46100 Spain

**Keywords:** Disability, Caregivers, Burden, Meaning in life, Loneliness, Depression

## Abstract

**Background:**

Family caregivers of individuals with disabilities often experience a significant caregiver burden, which has been associated with higher levels of depressive symptomatology. However, limited research has examined the role of moderating variables such as unwanted loneliness and meaning in life in this context. This study aims to analyze the presence of depressive symptoms among female informal caregivers and the modulating factors involved, highlighting meaning in life as a key psychological variable in the caregiving process.

**Methods:**

The sample consisted of 104 informal caregivers of individuals with disabilities, all of whom were women. Individual interviews were conducted, during which participants completed an assessment protocol comprising a sociodemographic questionnaire and the following standardized instruments: the Zarit Caregiver Burden Interview, the UCLA Loneliness Scale, the Patient Health Questionnaire-9 (PHQ-9), and the Purpose in Life Scale (PIL-10).

**Results:**

Findings revealed that meaning in life partially mediates the relationship between caregiver burden, unwanted loneliness, and depressive symptoms, acting as a protective factor against emotional distress in informal caregivers.

**Conclusions:**

Our results suggest that meaning in life is a key mediating variable in the link between caregiver burden, loneliness, and depression. We conclude that meaning in life is a relevant construct for understanding caregiver stress and should be considered in therapeutic interventions aimed at supporting informal caregivers.

Caring for a person with a disability is considered a chronic stressor due to the large number of demands that the caregiver must take on. Some of the demands of caregiving include constant supervision, assistance with self-care activities, aspects related to continence, communication, and mobility [1]. Caregivers must also deal with problematic behaviors and cover the numerous financial costs involved in caregiving.

Most children and young people with disabilities are cared for at home by their parents, which ultimately affects the health, finances, and well-being of the caregiver and their family. Caregivers experience psychological, emotional, social, and financial challenges due to caring for their family members with disabilities [2].

Caregiving situations are accompanied by holistic changes in the caregivers’ environment [3], who often suffer from overload, lack of information, financial stress, and a lower quality of life [4]. As a result of the limited opportunities to devote their time to leisure activities, the need to leave work (losing contact with colleagues), and changes in family dynamics [5], one aspect of caregivers’ lives that is severely affected is their social support network. In this regard, in many cases, the primary caregiver ends up becoming isolated, either due to a lack of time resulting from the responsibility of caregiving or due to feelings of misunderstanding from those around them [6, 7].

Throughout the life of the individual receiving care, parents are confronted with numerous stressors that can negatively impact both their physical and psychological health. Chronic stress is considered one of the most significant issues in this context [8–11]. This distress is often exacerbated by behavioral problems in the individual with a disability, the use of maladaptive coping strategies, or the presence of emotional difficulties [12].

In this regard, the literature has shown that parents of children with disabilities tend to experience higher rates of emotional disorders, such as anxiety and depression [6, 13], as well as elevated levels of emotional distress [14]. Specifically, recent studies have reported that mothers, in comparison to fathers, exhibit higher levels of psychological distress and depressive symptoms [15, 16].

Physically, caregivers often experience alterations in the cardiovascular, digestive, and immune systems [17], as well as other common symptoms such as back problems (muscle spasms), migraines, intestinal ulcers, arthritis or rheumatism, and high blood pressure [13].

Psychologically, caring for a child with a disability is associated with symptoms of anxiety, depression, overload, and loneliness, among others [10, 18–20], which often lead to poorer health and well-being [21]. It should be noted that, when analyzing the mental health of the caregiver at a more specific level, anxiety and depression have been given a relevant role [22, 23].

Some variables may play a modulating role, such as loneliness and meaning in life. Although loneliness has been explored in a variety of different populations, studies focusing on family caregivers are scarce [24–27], and few of them have examined loneliness in caregivers of children and young people with disabilities.

There is little evidence about the prevalence of loneliness in caregivers. In Spain, prevalence rates ranging from 25.6% to 30.5% have been reported [28]. The numerous demands faced by caregivers of children with disabilities can lead to feelings of loneliness, as other activities that promote socialization may be relegated to the background [29]. This can have negative consequences for the health of caregivers, including sleep problems, psychological stress, and an increased risk of early mortality [30].

The task of caregiving is mostly carried out by mothers who assume the role of primary caregiver and who, due to the high number of demands involved in caring for their child, end up having to devote themselves entirely to the caregiving process. This leads to less participation in leisure activities and a restructuring of their social networks, which can be associated with higher levels of loneliness [6, 31]. On the other hand, the caregiving situation itself can change the dynamics of the couple, as parents may differ in their style of coping with and managing the disease, creating conflict within the couple [10].

In addition, to cope with the task of caregiving, many caregivers lose or reduce their working hours, which, according to the literature, can also contribute to feelings of loneliness [32]. Work, in addition to being a way to earn a salary and gain social prestige, is also a source of participation in social life.

Feelings of loneliness in caregivers are associated with higher levels of anxiety, depression, and fatigue, among other things [33]. Previous studies show a greater significance in the relationship between feelings of loneliness and depression in caregivers compared to non-caregivers [34]. Due to the demands of caregiving and the lack of other types of support, many caregivers face problems of social isolation and withdrawal from social activities, which lead to increased feelings of loneliness.

Losses associated with caregiving can in turn influence losses of personal skills and resources necessary to initiate and/or maintain social support networks. Difficulty accessing this type of support often manifests itself in emotional and physical distress and feelings of lack of support, loneliness, or vulnerability [35]. In this sense, the primary caregiver often ends up isolating themselves from their network of friends and even family due to the responsibility of caregiving, whether due to lack of time or feelings of misunderstanding from those around them [6, 7].

While many studies have focused on interventions to reduce the burden on caregivers and improve their quality of life [36, 37], few have investigated the impact of loneliness on caregivers [38]. Many caregivers are at greater risk of suffering from a burden due to their difficulty in accepting help or denial of the need for services, which hinders the implementation of interventions [39] that could improve psychological well-being and reduce caregiver overload [40].

On the other hand, purpose in life, understood as the perception that life has direction, intentionality, and meaning, has become particularly relevant in the field of mental health and well-being in recent decades [41].

Emotional isolation creates situations of hopelessness within the family and aggravates the socio-emotional state of caregivers [18, 42]. The meaning of life has been identified as a protective factor against conditions of psychological and physiological vulnerability [43]. In this context, the role of informal caregiver poses emotional and physical demands that can directly affect the experience of meaning in life [44, 45].

Interestingly, some studies have revealed that, despite the strain of caregiving, certain caregivers may experience high levels of life purpose [44]. Likewise, recent research has shown that life purpose has a reciprocal relationship with the caregiver’s overall health and well-being [46, 47].

Finally, it has been noted that meaning in life enhances positive psychological resources, such as optimistic beliefs or constructive worldviews, which promote resilience in the face of adverse events [48]. This evidence supports the importance of addressing purpose in life as a key variable in the well-being of caregivers, especially in emotionally demanding contexts.

The purpose of this study was to examine the impact of caregiver burden, loneliness, and meaning in life on depressive symptoms among caregivers of individuals with disabilities. In addition, the study explored whether meaning in life mediated the relationship between caregiver burden and depression, as well as between loneliness and depression.

## Method

A cross sectional, observational, prospective study was developed to assess the relation between burden, loneliness, meaning in life, and depression in caregivers of people with disabilities. The study followed the recommendations of the Declaration of Helsinki and the anonymity and privacy of participants was ensured in the whole process.

As inclusion and exclusion criteria, participants were required to be adult women serving as the primary caregiver of a family member with a disability for at least 12 months. The rational for this criterion is that women are usually the ones taking the role of caregiver of the person with the disability [6, 31]. Individuals with difficulties understanding or responding to the questionnaire were excluded from the study. This was done to ensure reliability of the data and minimize potential sources of measurement error.

### Instruments

Revised UCLA Loneliness Scale [49, 50]. The scale consists of 20 items, each rated on a four-point Likert scale (1 = never, 4 = always). Total scores range from 20 to 80, with higher scores indicating greater levels of loneliness. The scale demonstrates high internal consistency, with a Cronbach’s alpha coefficient of 0.94. For this sample, the internal consistency of the UCLA scores were 0.92 for McDonald’s ω and 0.90 for McDonald’s ω.

The Zarit Caregiver Burden Interview [51, 52] is a widely used self-report instrument for assessing the perceived burden of caregivers. The version used in this study consists of 22 items; each rated on a five-point Likert scale from 1 (never) to 5 (nearly always). The scale measures the extent to which caregiving affects physical, psychological, social, and economic well-being, including leisure activities, social relationships, intimacy, and autonomy.

Cut-off scores for interpreting the burden scores were taken from a previous Spanish validation study [52], a cut-off score of 47 differentiated between no burden and mild burden (specificity = 84.4%, sensitivity = 85.1%), while a cut-off of 56 distinguished mild from severe burden (specificity and sensitivity = 93.3%). The instrument showed good reliability (test-retest = 0.86) and high internal consistency (Cronbach’s α = 0.91). For the current study, the internal consistency of the scores were McDonald’s ω = 0.92 and McDonald’s ω = 0.91.

The Patient Health Questionnaire-9 (PHQ-9) [53, 54] is a screening tool for detecting depressive symptoms based on the DSM-IV diagnostic criteria for major depressive disorders. It consists of 9 items assessing the frequency of symptoms over the past two weeks using a four-point Likert scale. Total scores range from 0 to 27, allowing classification of depression severity. The Spanish version has demonstrated good internal consistency (Cronbach’s α = 0.89). As for the reliability of the scores of PHQ-9 in this sample, McDonald’s ω = 0.91 and McDonald’s ω = 0.91.

The Purpose-in-Life Test–10 (PIL-10) [55] is a short form of the original Purpose-in-Life Test [56] designed to assess individuals’ perceived sense of meaning in life. It consists of 10 items rated on a 7-point Likert scale (1 to 7, with 4 as a neutral midpoint). The items assess dimensions such as enthusiasm for life, goal clarity, daily engagement, appreciation of life, and the presence of life purpose. Total scores range from 10 to 70, with higher scores indicating greater perceived purpose in life. The Spanish version shows good internal consistency (Cronbach’s α = 0.86), and reliability in this study was even higher (α = 0.92). The internal consistency of the scores of the participants of this study showed good internal consistency, McDonald’s ω = 0.88 and McDonald’s ω = 0.88.

#### Sociodemographic questionnaire

A sociodemographic questionnaire specifically designed for this study was used to collect background information on both the caregiver and the person with a disability. Participants were asked to report on the employment, educational status, marital status and age of the caregiver, and the age of the person and diagnosis of the person with the disability.

### Procedure

The study was approved by an Institutional Review Board (project code: blinded for review purposes, included in cover letter). Participants were recruited through collaborating centers across the Valencian Community, which provided contact information for individuals interested in participating. These centers belong to network of association which served a total of 350 individuals with disabilities. A telephone screening interview was conducted to explain the study and verify eligibility criteria. A total of 170 centers usuaries were contacted and invited to attend an in-person session at the collaborating centers, during which all study measures were administered. Informed consent was obtained prior to data collection for a total of 104 participants, for an attrition rate of 38,82%. To ensure anonymity and data confidentiality, each participant was assigned a research code, and all personally identifying information was removed from the dataset.

### Data analysis

The data analysis statistical software Jasp Version 0.19.3 [57] was used to run the analysis. Descriptive statistics were calculated to describe the sample and the mean scores and standard deviations of participants on the measurements. To identify the changes on the depressions scores depending on the 1-point variations on the caregiver burden, loneliness, and purpose in life scores, a linear regression analysis was performed. Before preforming the liner regression analysis, different assumptions were checked to ensure the adequacy of the data to run the analysis. First, the Durbin-Watson test [58, 59] was used to check for autocorrelation among the regression residuals, value close to 2 indicate no autocorrelation, for this regression the statistic was 1.19, *p* = .01, indicating the presence of a statically significant positive autocorrelation of the residuals of the regression. Second, the homoscedasticity was check using a Residuals vs. Predicted values graph, the order of the data points indicated adequacy of the data to run the analysis. As for, multicollinearity, Pearson correlation analyses were run, correlation indexes were interpreted following [60] recommendations: 0.20 > *r* < .50 = small correlation; 0.50 > *r* < .80 = moderate; and *r* > .80 = strong correlation. All correlation indexes among the predictor variables were below 0.70, correlation values ranged from − 0.59 to 0.63. Prior to conducting the linear regression analysis, the tolerance and variance inflation factor (VIF) were examined to assess multicollinearity among the independent variables. A tolerance value above 0.1 and a VIF below 10 were considered indicative of acceptable levels of multicollinearity [61]. All variables met these criteria, tolerance values ranged from 0.56 to 0.69 and VIF values ranged from 1.45 to 1.79, ensuring the appropriateness of their inclusion in the regression model.

Finally, a mediation analysis was carried out to identify the effects of caregiver burden (X_1_) through purpose in life (M_1_) on depression (Y_1_), and of loneliness (X_2_) through purpose in life (M_1_) on depression (Y_1_). This analysis was, also, run in JASP Version 0.19.03 [57], it was utilized the maximum likelihood estimator and delta method for standard errors, with a 95% confidence interval and a Bias-Corrected Percentile Bootstrap of 5000 replications. All variables were standardized prior to analysis.

## Results

### Participants

A total of 104 primary caregivers of individuals with disabilities participated in the study. All participants were women, with a mean age of 48.24 years (SD = 9.28, range = 25–75 years). The children or dependents with disabilities they cared for had a mean age of 15.39 years (SD = 9.64, range = 1–40 years).

Regarding marital status, most participants were married or in a domestic partnership (69.2%), followed by single (10.6%), divorced (9.6%), widowed (5.8%), and separated (4.8%). In terms of educational background, 39.4% had completed secondary education, 32.7% had a university degree, 16.3% had completed primary school, and 11.5% had postgraduate studies. Employment status was relatively balanced: 36.5% reported not working, while 31.7% were employed part-time and another 31.7% full-time.

The children and dependents being cared for had a variety of diagnoses. The most prevalent conditions were autism spectrum disorder (39.4%), intellectual disability (23.1%), and rare diseases (14.4%). Other diagnoses included Down syndrome (3.8%), cerebral palsy (5.8%), and various low-frequency syndromes such as Fragile X, Phelan McDermid, fetal alcohol syndrome, and epilepsy (Table 1).

**Table 1:** Sociodemographic and diagnostic characteristics of the sample

Variables	Frequency (%)
Marital Status	
Married/Domestic Partnership	72 (69.2%)
Single	11 (10.6%)
Divorced	10 (9.6%)
Widowed	6 (5.8%)
Separated	5 (4.8%)
Education	
Secondary	41 (39.4%)
University	34 (32.7%)
Primary	17 (16.3%)
Postgraduate	12 (11.5%)
Employment	
Not working	38 (36.5%)
Part-time	33 (31.7%)
Full-time	33 (31.7%)
Diagnosis	
Autism Spectrum Disorder	41 (39.4%)
Intellectual Disability	24 (23.1%)
Rare Disease	15 (14.4%)
Cerebral Palsy	6 (5.8%)
Down Syndrome	4 (3.8%)
Fragile X Syndrome	3 (2.9%)
ADHD	2 (1.9%)
Other (e.g., epilepsy, SAF, OCD, etc.)	9 (8.7%)

A Pearson correlation analysis was used to explore the relation among caregiver burden (ZARIT), loneliness (UCLA), purpose in life (PIL), and depressive symptoms (PHQ). Results revealed significant and moderate to strong associations between all variables. Loneliness was positively correlated with depression, *r* =.629, *p* <.001, and caregiver burden, *r* =.614, *p* <.001. Depression was also positively associated with caregiver burden, *r* =.580, *p* <.001. On the other hand, purpose in life showed significant negative correlations with loneliness (*r* = −.513, *p* <.001), depression (*r* = −.588, *p* <.001), and caregiver burden (*r* = −.485, *p* <.001). These findings indicate that higher levels of loneliness and caregiver burden are associated with greater depressive symptoms, whereas a stronger sense of purpose in life is associated with lower levels of loneliness, burden, and depression.

A linear regression analysis was conducted to examine the extent to which caregiver burden (ZARIT), loneliness (UCLA), and purpose in life (PIL) predict depressive symptoms (PHQ) among primary caregivers. The regression model was statistically significant (*F* (3, 100) = 36.33, *p* <.001), accounting for 52.1% of the variance in depression (PHQ) scores (*R*² = 0.521). All predictors contributed significantly to the model. Specifically, higher purpose in life predicted lower depression scores (*β* = −0.307, *p* <.001), whereas greater loneliness (*β* = 0.332, *p* <.001) and caregiver burden (*β* = 0.227, *p* =.014) were associated with increased depressive symptoms.

Moreover, the unstandardized coefficients (b values) from the regression model allow for interpretation of changes in depressive symptoms (PHQ score) in real units of measurement. The results indicate that for every 1-point increase in caregiver burden (ZARIT score), the depression score increases by 0.095 points. For example, a 10-point increase in ZARIT scores predicts a 0.95-point increase in PHQ scores. For every 1-point increase in loneliness (UCLA score), the depression score increases by 0.338 points. Conversely, for every 1-point increase in purpose in life (PIL score), the depression score decreases by 0.405 points. These results indicate that loneliness exerts the strongest positive influence on depression, followed by caregiver burden. Meanwhile, purpose in life plays a protective role by significantly reducing depressive symptoms (Table 2).

**Table 2:** Descriptive statistics and regression coefficients predicting depression

Variables	M (SD)	b	SE	β	t	*p*	95% CI LL-UL
PIL	19.27 (5.39)	−0.405	0.110	−0.307	−3.691	< 0.001	−0.623 – −0.187
UCLA	66.40 (6.98)	0.338	0.094	0.332	3.604	< 0.001	0.152–0.525
ZARIT	61.88 (16.97)	0.095	0.038	0.227	2.506	0.014	0.020–0.170
PHQ	11.72 (7.10)						

*Note. N* = 104. *ZARIT =* Caregiver burden. *UCLA =* Loneliness. *PHQ =* Depression. *PIL =* Purpose in Life

**Fig. 1:**
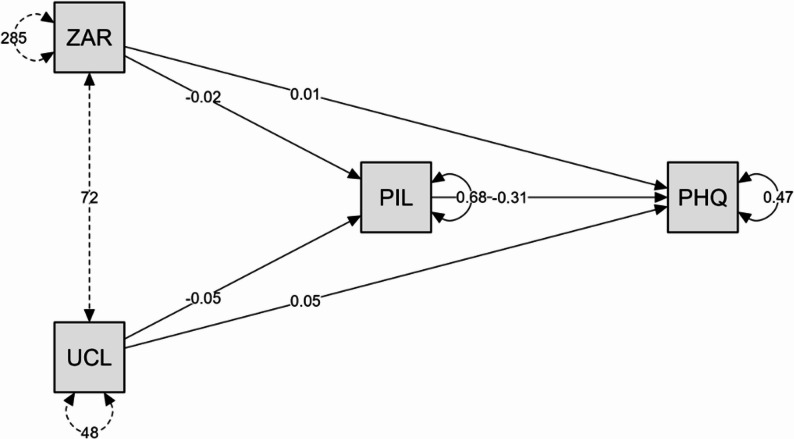
Mediation model to determine the effect of caregiver burden and loneliness on depression through purpose in life

A mediation analysis was conducted to examine whether the relationship between caregiver burden (ZARIT, X_1_) and loneliness (UCLA, X_2_) on depressive symptoms (PHQ, Y_1_) was mediated by purpose in life (PIL, M_1_). The analysis utilized the maximum likelihood estimator and delta method for standard errors, confidence intervals of 95%, and Bootstrap of 5000 replications. Results indicate both direct and indirect effects, supporting a partial mediation model (Fig. 1).

The total effects were also significant for both caregiver burden (β = 0.018, *p* <.001) and loneliness (*β* = 0.063, *p* <.001). Notably, the path coefficients indicate that caregiver burden (β = −0.016, *p* =.008) and loneliness (*β* = −0.050, *p* <.001) were negatively associated with purpose in life, while purpose in life was negatively associated with depression (*β* = −0.307, *p* <.001). The model accounted for 52.1% of the variance in depression and 31.0% in purpose in life, indicating a substantial explanatory power (Table XX). In other words, caregiver burden and loneliness have an impact on depression and purpose in life. Direct effects showed that both caregiver burden (*β* = 0.013, *p* =.011,) and loneliness (*β* = 0.048, *p* <.001) significantly predicted depressive symptoms. (Table 3).

**Table 3:** Path coefficients for Direct, indirect and total effects

Paths	Estimate (β)	SE	Z	*p*	95% CI LL-UL
Direct effects					
ZARIT → PHQ	0.013	0.005	2.556	0.011	0.002–0.025
UCLA → PHQ	0.048	0.013	3.676	< 0.001	0.017–0.074
ZARIT → PIL	−0.016	0.006	−2.645	0.008	−0.003 – −0.002
UCLA → PIL	−0.050	0.015	−3.346	< 0.001	−0.083 – −0.014
PIL → PHQ	−0.307	0.082	−3.764	< 0.001	−0.472 – −0.121
Indirect effects					
ZARIT → PIL → PHQ	0.005	0.002	2.164	0.030	0.001–0.012
UCLA → PIL → PHQ	0.015	0.006	2.501	0.012	0.005–0.031
Total effects					
ZARIT → PHQ	0.018	0.005	3.393	< 0.001	0.008–0.029
UCLA → PHQ	0.063	0.013	4.788	< 0.001	0.032–0.090

*Note. N* = 104. *ZARIT =* Caregiver burden. *UCLA =* Loneliness. *PHQ =* Depression. *PIL =* Purpose in Life

Indirect effects on depressive symptoms via purpose in life were also statistically significant for both variables: caregiver burden (*β* = 0.005, *p* =.030) and loneliness (*β* = 0.015, *p* =.012), suggesting that purpose in life partially mediates the impact of burden and loneliness on depression. The total effect of caregiver burden (0.018) is larger than the direct effect (0.013), meaning that part of the effect of caregiver burden on depression is mitigated through purpose in life. Specifically, purpose in life reduces the effect of caregiver burden on depression, acting as a buffering or protective factor. Similarly, the total effect of loneliness (0.063) is larger than the direct effect (0.048), indicating that purpose in life also mediates part of the effect of loneliness on depression. Once again, purpose in life reduces the total impact of loneliness on depressive symptoms.

## Discussion

The results of this study suggest the existence of statistically significant relationships between caregiver burden (ZARIT), social loneliness (UCLA), meaning in life (PIL), and depressive symptoms (PHQ), as well as the mediating role of meaning in life in these relationships. In line with previous research [62, 63], both caregiver burden and perceived loneliness are positively associated with levels of depression, while meaning in life is negatively correlated with all these constructs.

First, the positive correlation between caregiver burden and depression, widely documented in the literature [64–66], is confirmed once again. Caregivers who report greater overload tend to have higher levels of emotional distress, which could be explained by the accumulation of physical, emotional, and economic demands that negatively impact their mental health.

Likewise, a positive and significant relationship between loneliness and depression was found. This bidirectional association has been supported by numerous studies and meta-analyses [67–69], which show that loneliness not only predicts the onset of depressive symptoms, but that depression itself can intensify the perception of isolation and social disconnection.

A particularly relevant finding of this study is the mediating role of meaning in life (MIL) in the relationship between the study variables (caregiver burden and loneliness) and depression. The results indicate that high levels of perceived caregiver burden and loneliness are associated with a decrease in meaning in life. Furthermore, when meaning in life was included as a mediating variable between loneliness, burden, and depression, it was found that meaning in life (PIL) reduces the effect of loneliness and burden on depression. This mediation was statistically significant, reinforcing the hypothesis that meaning in life acts as a protective factor against emotional deterioration in caregivers, in line with previous research [70, 71].

The negative relationship between meaning in life and depression is consistent with theoretical models that highlight the importance of life purpose in emotional regulation and resilience [63]. In chronic care settings, a greater sense of meaning in life is associated with better adaptation, less stress, and greater subjective satisfaction, which buffers the impact of stressors such as burden or loneliness [72, 73].

On the other hand, the negative correlation between meaning in life and loneliness supports the idea that these constructs are interdependent. A caregiver who finds meaning in their role may perceive less isolation and may function as an internal resource for coping with the adversities of caregiving in a more positive way.

Loneliness can erode life purpose, promoting hopelessness [63]. This finding is especially relevant in psychological interventions, as it suggests that fostering meaning in life could reduce both the perception of loneliness and depressive symptoms.

The present study highlights the relevance of meaning in life in the caregiving process and the need for studies aimed at its analysis and intervention. From an applied perspective, the results obtained underscore the need to implement intervention programs focused on strengthening life purpose in caregivers. Interventions based on logotherapy and acceptance and commitment therapy (ACT) could be effective in mitigating depressive symptoms and improving the quality of life of this vulnerable group [39].

However, this study has certain limitations. First, we recognize that the findings of this research cannot be generalized to the experience of caregivers of people with disabilities. Our analyses provided information on the relationship between loneliness, overload, sense of meaning in life, and emotional health in a sample of female caregivers in different metropolitan areas of the city of Valencia. It would be relevant to confirm these results in larger samples that include male caregivers.

Second, the cross-sectional design prevents us from establishing definitive causal relationships between the variables. Future research should incorporate longitudinal or experimental designs that allow for the evaluation of the temporal evolution of the model and the effectiveness of interventions aimed at life purpose in order to understand how loneliness and overload influence emotional well-being and to what extent caregivers’ sense of meaning in life can be a modulating variable over time and as the years of dedication to the caregiving process progress. Furthermore, qualitative and mixed-method approaches can contribute to a greater understanding of explanatory models of stress.

Likewise, contextual variables such as perceived social support and characteristics of care (duration, involvement, among others), as well as the support needs of the person being cared for, could moderate the observed effects and should be included in future analytical models.

Thirdly, given that the study was based on self-reports, there may have been a social desirability bias. In this regard, participants were aware that the study focused on aspects related to caregiver burden, which may have limited participation to only those caregivers interested in this topic.

In conclusion, the present study provides empirical evidence of the central role that meaning in life plays as a mediator between caregiver burden, loneliness, and depression. The results of the regression and mediation analyses highlight the potential value of interventions aimed at reducing caregiver burden and loneliness. The findings underscore the importance of enhancing meaning in life, given its role as both a predictor of depressive symptoms and a mediator in the relation between caregiver burden, loneliness, and depression in caregivers of individuals with disabilities. Moreover, the role of loneliness is noteworthy, as there are few studies that analyze loneliness, which is underrepresented in explanatory models of stress that address the caregiving process. These findings not only enrich the theoretical understanding of psychological distress in caregivers, but also offer practical implications for the design of clinical and community interventions aimed at promoting emotional well-being in this at-risk group.

## Data Availability

The dataset used in this study is publicly available through ZENODO and the following link: [https://doi.org/10.5281/zenodo.16530131](https:/doi.org/10.5281/zenodo.16530131).

## References

[1] Patel DR, Cabral MD, Ho A, Merrick J. A clinical primer on intellectual disability. Transl Pediatr [Internet]. 2020;9(Suppl 1):S23–35. 10.21037/tp.2020.02.02. Available on:. 32206581 10.21037/tp.2020.02.02 PMC7082244

[2] Fernández-Ávalos MI, Pérez-Marfil MN, Ferrer-Cascales R, Cruz-Quintana F, Clement-Carbonell V, Fernández-Alcántara M. Quality of life and concerns in parent caregivers of adult children diagnosed with intellectual disability: A qualitative study. Int J Environ Res Public Health [Internet]. 2020;17(22):8690. Available on: 10.3390/ijerph17228690 10.3390/ijerph17228690 PMC7709017 33238511

[3] Rodakowski J, Skidmore ER, Rogers JC, Schulz R. Role of social support in predicting caregiver burden. Arch Phys Med Rehabil [Internet]. 2012;93(12):2229–36. 10.1016/j.apmr.2012.07.004. Available on:. 22824248 10.1016/j.apmr.2012.07.004 PMC3508254

[4] Samadi H, Samadi SA. Understanding different aspects of caregiving for individuals with autism spectrum disorders (ASDs) a narrative review of the literature. Brain Sci [Internet]. 2020;10(8):557. 10.3390/brainsci10080557. Available on:. 32824109 10.3390/brainsci10080557 PMC7463436

[5] Lederman VRG, Alves B, dos S, Negrão J, Schwartzman JS, D’Antino MEF, Brunoni D. Divorce in families of children with down syndrome or Rett syndrome. Cien Saude Colet [Internet]. 2015;20(5):1363–9. 10.1590/1413-81232015205.13932014. Available on:. 26017939 10.1590/1413-81232015205.13932014

[6] Kolemen AB, Akyuz E, Toprak A, Deveci E, Yesil G. Evaluation of the parents’ anxiety levels before and after the diagnosis of their child with a rare genetic disease: the necessity of psychological support. Orphanet J Rare Dis. 2021;16(1):402. Available on: 10.1186/s13023-021-02046-2 10.1186/s13023-021-02046-2 PMC8480067 34583726

[7] Smits RM, Vissers E, Te Pas R, Roebbers N, Feitz WFJ, van Rooij IALM et al. Common needs in uncommon conditions: a qualitative study to explore the need for care in pediatric patients with rare diseases. Orphanet J Rare Dis. 2022;17(1):153. Available on: 10.1186/s13023-022-02305-w 10.1186/s13023-022-02305-w PMC8981675 35379257

[8] Graungaard AH, Skov L. Why do we need a diagnosis? A qualitative study of parents’ experiences, coping and needs, when the newborn child is severely disabled. Child Care Health Dev [Internet]. 2007;33(3):296–307. 10.1111/j.1365-2214.2006.00666.x. Available on:. 17439444 10.1111/j.1365-2214.2006.00666.x

[9] Lee JCK. So rare, who cares? A study on stress and difficulties encountered by the parents of children with a rare disease in Hong Kong. Hong Kong J Soc Work [Internet]. 2020;54(01n02):13–30. 10.1142/s0219246220000042. Available on:.

[10] Picci RL, Oliva F, Trivelli F, Carezana C, Zuffranieri M, Ostacoli L, et al. Emotional burden and coping strategies of parents of children with rare diseases. J Child Fam Stud [Internet]. 2015;24(2):514–22. 10.1007/s10826-013-9864-5. Available on:.

[11] van Oers HA, Haverman L, Limperg PF, van Dijk-Lokkart EM, Maurice-Stam H, Grootenhuis MA. Anxiety and depression in mothers and fathers of a chronically ill child. Matern Child Health J [Internet]. 2014;18(8):1993–2002. 10.1007/s10995-014-1445-8. Available on:. 24791971 10.1007/s10995-014-1445-8

[12] Minnes P, Perry A, Weiss JA. Predictors of distress and well-being in parents of young children with developmental delays and disabilities: the importance of parent perceptions: parent well-being. J Intellect Disabil Res [Internet]. 2015;59(6):551–60. 10.1111/jir.12160. Available on:. 25169777 10.1111/jir.12160

[13] Gallagher S, Hannigan A. Depression and chronic health conditions in parents of children with and without developmental disabilities: the growing up in Ireland cohort study. Res Dev Disabil [Internet]. 2014;35(2):448–54. 10.1016/j.ridd.2013.11.029. Available on:. 24361813 10.1016/j.ridd.2013.11.029

[14] Fitzgerald J, Gallagher L. Parental stress and adjustment in the context of rare genetic syndromes: A scoping review. J Intellect Disabil [Internet]. 2021;26(2):1744629521995378. 10.1177/1744629521995378. Available on:. 10.1177/1744629521995378 PMC9168905 33866895

[15] van Niekerk K, Stancheva V, Smith C. Caregiver burden among caregivers of children with autism spectrum disorder. S Afr J Psychiatr [Internet]. 2023. 10.4102/sajpsychiatry.v29i0.2079. Available on:. 29:2079. 37928940 10.4102/sajpsychiatry.v29i0.2079 PMC10623632

[16] Vats T, Sinha AK, Arun P. Gender differences in caregiving: an exploration among families of individuals with autism spectrum disorder. Autism Spectr Disorder Frontier Anthropol. 2023;12:19–31.

[17] Cohn LN, Pechlivanoglou P, Lee Y, Mahant S, Orkin J, Marson A et al. Health outcomes of parents of children with chronic illness: A systematic review and meta-analysis. J Pediatr. 2020; 218:166–177.e2. Available on: 10.1016/j.jpeds.2019.10.068 10.1016/j.jpeds.2019.10.068 31916997

[18] Hassall S, Smith DM, Rust S, Wittkowski A. A systematic review and integrative sequential explanatory narrative synthesis: the psychosocial impact of parenting a child with a lysosomal storage disorder. J Inherit Metab Dis [Internet]. 2022;45(3):406–16. 10.1002/jimd.12482. Available on:. 35124835 10.1002/jimd.12482 PMC9305282

[19] Schiller VF, Dorstyn DS, Taylor AM. The protective role of social support sources and types against depression in caregivers: A meta-analysis. J Autism Dev Disord [Internet]. 2021;51(4):1304–15. 10.1007/s10803-020-04601-5. Available on:. 32683544 10.1007/s10803-020-04601-5

[20] Pelentsov LJ, Fielder AL, Laws TA, Esterman AJ. The supportive care needs of parents with a child with a rare disease: results of an online survey. BMC Fam Pract [Internet]. 2016;17:88. 10.1186/s12875-016-0488-x. Available on:. 27439905 10.1186/s12875-016-0488-x PMC4955113

[21] Barker ET, Greenberg JS, Seltzer MM, Almeida DM. Daily stress and cortisol patterns in parents of adult children with a serious mental illness. Health Psychol. 2012;31(1):130–4. 10.1037/a0025325. 21895369 10.1037/a0025325 PMC3254790

[22] Barker ET, Hartley SL, Seltzer MM, Floyd FJ, Greenberg JS, Orsmond GI. Trajectories of emotional well-being in mothers of adolescents and adults with autism. Dev Psychol. 2011;47(2):551–61. 10.1037/a0021268. 21171753 10.1037/a0021268 PMC3074104

[23] Phetrasuwan S, Shandor Miles M. Parenting stress in mothers of children with autism spectrum disorders. J Spec Pediatr Nurs. 2009;14(3):157–65. 10.1111/j.1744-6155.2009.00188.x. 19614824 10.1111/j.1744-6155.2009.00188.x

[24] Chukwuorji JC, Amazue LO, Ekeh OH. Loneliness and psychological health of orthopaedic patients’ caregivers: does gender make a difference? Psychol Health Med. 2017;22(4):501–6. 10.1080/13548506.2016.1173711. 28114810 10.1080/13548506.2016.1173711

[25] Ekwall AK, Sivberg B, Hallberg IR. Loneliness as a predictor of quality of life among older caregivers. J Adv Nurs. 2005;49(1):23–32. 10.1111/j.1365-2648.2004.03260.x. 15610378 10.1111/j.1365-2648.2004.03260.x

[26] McRae C, Fazio E, Hartsock G, Kelley L, Urbanski S, Russell D. Predictors of loneliness in caregivers of persons with Parkinson’s disease. Parkinsonism Relat Disord. 2009;15(8):554–7. 19251464 10.1016/j.parkreldis.2009.01.007 PMC2749069

[27] Vasileiou K, Barnett J, Barreto M, Vines J, Atkinson M, Lawson S et al. Experiences of loneliness associ-ated with being an informal caregiver: A qualitative investigation. Front Psychol. 2017;8. Available on: 10.3389/fpsyg.2017.00585 10.3389/fpsyg.2017.00585 PMC5395647 28469589

[28] Huertas-Domingo C, Márquez-González M, Cabrera I, Barrera-Caballero S, Pedroso-Chaparro MDS, Romero-Moreno R, et al. Sociocultural influences on the feeling of loneliness of family caregivers of people with dementia: the role of kinship. Int J Environ Res Public Health. 2021;18(9):4700. 10.3390/ijerph18094700. 33925135 10.3390/ijerph18094700 PMC8125119

[29] Vitaliano PP, Murphy M, Young HM, Echeverria D, Borson S. Does caring for a spouse with dementia promote cognitive decline? A hypothesis and proposed mechanisms: caregiving and cognitive decline. J Am Geriatr Soc. 2011;59(5):900–8. 10.1111/j.1532-5415.2011.03368.x. 21568959 10.1111/j.1532-5415.2011.03368.x

[30] Hawkley LC, Capitanio JP. Perceived social isolation, evolutionary fitness and health outcomes: a lifespan approach. Philos Trans R Soc Lond B Biol Sci [Internet]. 2015;370(1669):20140114. 10.1098/rstb.2014.0114. Available on:. 25870400 10.1098/rstb.2014.0114 PMC4410380

[31] Kovaleva M, Spangler S, Clevenger C, Hepburn K. Chronic stress, social isolation, and perceived loneliness in dementia caregivers. J Psychosoc Nurs Ment Health Serv [Internet]. 2018;56(10):36–43. 10.3928/02793695-20180329-04. Available on:. 29667698 10.3928/02793695-20180329-04

[32] Bremer P, Cabrera E, Leino-Kilpi H, Lethin C, Saks K, Sutcliffe C. Informal dementia care: consequencesfor caregivers’ health and health care use in 8 European countries. Health Policy. 2015;119(11):1459–71. 26518906 10.1016/j.healthpol.2015.09.014

[33] Jaremka LM, Andridge RR, Fagundes CP, Alfano CM, Povoski SP, Lipari AM, et al. Pain, depression, and fatigue: loneliness as a longitudinal risk factor. Health Psychol [Internet]. 2014;33(9):948–57. 10.1037/a0034012. Available on:. 23957903 10.1037/a0034012 PMC3992976

[34] Beeson RA. Loneliness and depression in spousal caregivers of those with alzheimer’s disease versus non-caregiving spouses. Arch Psychiatr Nurs [Internet]. 2003;17(3):135–43. 10.1016/s0883-9417(03)00057-8. Available on:. 12840806 10.1016/s0883-9417(03)00057-8

[35] Anderson M, Elliott EJ, Zurynski YA. Australian families living with rare disease: experiences of diagnosis, health services use and needs for psychosocial support. Orphanet J Rare Dis. 2013;8(1):22. Available on: 10.1186/1750-1172-8-22 10.1186/1750-1172-8-22 PMC3599672 23398775

[36] Adelman RD, Tmanova LL, Delgado D, Dion S, Lachs MS. Caregiver burden: a clinical review. JAMA. 2014;311(10):1052. 10.1001/jama.2014.304. 24618967 10.1001/jama.2014.304

[37] Jensen M, Agbata IN, Canavan M, McCarthy G. Effectiveness of educational interventions for informal caregivers of individuals with dementia residing in the community: systematic review and meta-analysis of randomised controlled trials: dementia caregiver education: systematic review and meta-analysis. Int J Geriatr Psychiatry [Internet]. 2015;30(2):130–43. 10.1002/gps.4208. Available on:. 25354132 10.1002/gps.4208

[38] Velloze IG, Jester DJ, Jeste DV, Mausbach BT. Interventions to reduce loneliness in caregivers: an integrative review of the literature. Psychiatry Res [Internet]. 2022;311(114508):114508. 10.1016/j.psychres.2022.114508. Available on:. 35334424 10.1016/j.psychres.2022.114508

[39] Brodaty H, Thomson C, Thompson C, Fine M. Why caregivers of people with dementia and memory loss don’t use services. Int J Geriatr Psychiatry. 2005;20(6):537–46. 10.1002/gps.1322. 15920707 10.1002/gps.1322

[40] Ragni B, Boldrini F, Mangialavori S, Cacioppo M, Capurso M, De Stasio S. The efficacy of parent training interventions with parents of children with developmental disabilities. Int J Environ Res Public Health. 2022;19(15):9685. 10.3390/ijerph19159685. 35955038 10.3390/ijerph19159685 PMC9367974

[41] Ryff CD, Singer B. Psychological well-being: meaning, measurement, and implications for psychotherapy research. Psychother Psychosom. 1996;65(1):14–23. 10.1159/000289026. 8838692 10.1159/000289026

[42] Carona C, Silva N, Crespo C, Canavarro MC. Caregiving burden and parent-child quality of life outcomes in neurodevelopmental conditions: the mediating role of behavioral disengagement. J Clin Psychol Med Settings. 2014;21(4):320–8. 10.1007/s10880-014-9412-5. 25228103 10.1007/s10880-014-9412-5

[43] Cohen R, Bavishi C, Rozanski A. Purpose in life and its relationship to all-cause mortality and cardiovascular events: a meta-analysis: a meta-analysis. Psychosom Med. 2016;78(2):122–33. 10.1097/PSY.0000000000000274. 26630073 10.1097/PSY.0000000000000274

[44] Rhoades DR, McFarland KF. Purpose in life and self-actualization in agency-supported caregivers. Community Ment Health J. 2000;36(5):513–21. 10.1023/a:1001915831177. 10994684 10.1023/a:1001915831177

[45] Kroenke K, Spitzer RL, Williams JB. The PHQ-9: validity of a brief depression severity measure. J Gen Intern Med. 2001;16(9):606–13. 10.1046/j.1525-1497.2001.016009606.x. 11556941 10.1046/j.1525-1497.2001.016009606.x PMC1495268

[46] Yamamoto-Mitani N, Wallhagen MI. Pursuit of psychological well-being (ikigai) and the evolution of self-understanding in the context of caregiving in Japan. Cult Med Psychiatry. 2002;26(4):399–417. 10.1023/a:1021747419204. 12572767 10.1023/a:1021747419204

[47] Saylor A, Pavlovic M, Degroot N, Peeler L, Nelson A, Perrin KE. Feasibility of a multi-component strengths-building intervention for caregivers of persons with heart failure. J Appl Gerontol. 2023;42(12):2371–82. 37707361 10.1177/07334648231191595 PMC10840901

[48] Polenick CA, Kales HC, Birditt KS. Perceptions of purpose in life within spousal care dyads: associations with emotional and physical caregiving difficulties. Ann Behav Med [Internet]. 2018;52(1):77–87. 10.1093/abm/kax005. 10.1093/abm/kax005 PMC6361277 29538622

[49] Sutin AR, Luchetti M, Stephan Y, Sesker AA, Terracciano A. Propósito de vida, Mentalidad de estrés y estrés percibido: Prueba de Un Modelo de mediación. Pers Individ Dif. 2023;210. 10.1016/j.paid.2023.112227.

[50] Russell D, Peplau LA, Cutrona CE. The revised UCLA loneliness scale: concurrent and discriminant validity evidence. J Pers Soc Psychol. 1980;39(3):472–80. 10.1037//0022-3514.39.3.47. 10.1037//0022-3514.39.3.472 7431205

[51] Expósito F, Moya M. Validación de la UCLA Loneliness Scale en una muestra española. En: Loscertales-Abril E, Marín-Sánchez F, editores. Dimensiones psicosociales de la educación y de la comunicación. 1993. pp. 355–64.

[52] Zarit SH, Reever KE, Bach-Peterson J. Relatives of the impaired elderly, correlates of feelings of burden. Gerontologist. 1980;20:649–55. 7203086 10.1093/geront/20.6.649

[53] Martin M, Salvadó I, Nadal S, Miji LC, Rico JM, Lanz P. Adaptación Para Nuestro medio de La Escala de sobrecarga Del Cuidador (caregiver burden interview) de Zarit. Zarit Rev Gerontol. 1996;6:338–46.

[54] Diez-Quevedo C, Rangil T, Sanchez-Planell L, Kroenke K, Spitzer RL. Validation and utility of the patient health questionnaire in diagnosing mental disorders in 1003 general hospital Spanish inpatients. Psychosom Med [Internet]. 2001;63(4):679–86. 10.1097/00006842-200107000-00021. Available on:. 11485122 10.1097/00006842-200107000-00021

[55] García-Alandete J. Análisis factorial de una versión española de Purpose-In-Life Test, en función del género y edad. Pensam Psicol. 2014;12(1). Available on: 10.11144/javerianacali.ppsi12-1.afve

[56] Crumbaugh JC, Maholick LT. Manual de instrucciones Para El test de Propósito En La Vida. Munster: Psychometric Affiliates; 1969.

[57] JASP Team. (2024). JASP (Version 0.19.3)[Computer software].

[58] Durbin J, Watson GS. Testing for serial correlation in least squares regression: I. Biometrika [Internet]. 1950;37(3/4):409. Available on: 10.2307/2332391 14801065

[59] Durbin J, Watson GS. Testing for serial correlation in least squares regression. II Biometrika [Internet]. 1951;38(1/2):159. 10.2307/2332325. Available on:. 14848121

[60] Cohen J. Statistical power analysis for the behavioral sciences. 2nd ed. Hillsdale (NJ): Lawrence Erlbaum Associates; 1988.

[61] Kutner MH, Nachtsheim CJ, Neter J, Li W. Applied linear statistical models. 5th ed. New York (NY): McGraw-Hill/Irwin; 2005.

[62] Abbasi A, Mirhosseini S, Basirinezhad MH, Ebrahimi H. Relationship between caring burden and quality of life in caregivers of cancer patients in Iran. Support Care Cancer [Internet]. 2020;28(9):4123–9. 10.1007/s00520-019-05240-y. Available on:. 31872293 10.1007/s00520-019-05240-y

[63] Musich S, Wang SS, Kraemer S, Yeh CS. The association of psychological protective factors with caregiver mental health outcomes. Geriatr Nurs [Internet]. 2023;50:174–80. 10.1016/j.gerinurse.2023.01.020. Available on:. 36791542 10.1016/j.gerinurse.2023.01.020

[64] del-Pino-Casado R, Rodríguez Cardosa M, López-Martínez C, Orgeta V. The association between subjective caregiver burden and depressive symptoms in carers of older relatives: A systematic review and meta-analysis. PLoS One [Internet]. 2019;14(5):e0217648. 10.1371/journal.pone.0217648. Available on:. 31141556 10.1371/journal.pone.0217648 PMC6541277

[65] Pinquart M, Sörensen S. Associations of stressors and uplifts of caregiving with caregiver burden and depressive mood: A meta-analysis. J Gerontol B Psychol Sci Soc Sci [Internet]. 2003;58(2):P112–28. 10.1093/geronb/58.2.p112. Available on:. 12646594 10.1093/geronb/58.2.p112

[66] Lv R, Yang L, Li J, Kuang J, Zhou K, Xu M. Depression mediates the association between burden and quality of life in informal caregivers of stroke survivors: Meta-analytic structural equation modeling. Arch Phys Med Rehabil [Internet]. 2024;105(10):1961–70. 10.1016/j.apmr.2024.01.022. Available on:. 38367834 10.1016/j.apmr.2024.01.022

[67] Erzen E, Çikrikci Ö. The effect of loneliness on depression: A meta-analysis. Int J Soc Psychiatry [Internet]. 2018;64(5):427–35. 10.1177/0020764018776349. Available on:. 29792097 10.1177/0020764018776349

[68] Dunn C, Sicouri G. The relationship between loneliness and depressive symptoms in children and adolescents: A meta-analysis. Behav Change [Internet]. 2022;39(3):134–45. 10.1017/bec.2022.13. Available on:.

[69] Zhang Y, Kuang J, Xin Z, Fang J, Song R, Yang Y, et al. Loneliness, social isolation, depression and anxiety among the elderly in shanghai: findings from a longitudinal study. Arch Gerontol Geriatr [Internet]. 2023;110(104980):104980. 10.1016/j.archger.2023.104980. Available on:. 36863166 10.1016/j.archger.2023.104980

[70] Chang Y-P, Sessanna L, Seo YS. Perceived life meaning and purpose and its association with mental and physical health among family caregivers. Innov Aging [Internet]. 2021;5(Supplement1):797–8. 10.1093/geroni/igab046.2940. Available on:.

[71] Hua Z, Ma D. Purpose in life moderates the relationship between loneliness and caregiving stress among family caregivers of people with mental health problems. Arch Psychiatr Nurs [Internet]. 2024;49:99–105. 10.1016/j.apnu.2024.02.009. Available on:. 38734461 10.1016/j.apnu.2024.02.009

[72] Mwilambwe-Tshilobo L, Ge T, Chong M, Ferguson MA, Misic B, Burrow AL, et al. Loneliness and meaning in life are reflected in the intrinsic network architecture of the brain. Soc Cogn Affect Neurosci [Internet]. 2019;14(4):423–33. 10.1093/scan/nsz021. Available on:. 30924854 10.1093/scan/nsz021 PMC6523421

[73] Rha SY, Park Y, Song SK, Lee CE, Lee J. Caregiving burden and the quality of life of family caregivers of cancer patients: the relationship and correlates. Eur J Oncol Nurs [Internet]. 2015;19(4):376–82. 10.1016/j.ejon.2015.01.004. Available on:. 25795160 10.1016/j.ejon.2015.01.004

